# Predicting the outcome of competition when fitness inequality is variable

**DOI:** 10.1098/rsos.150274

**Published:** 2015-08-12

**Authors:** Michael T. Pedruski, Gregor F. Fussmann, Andrew Gonzalez

**Affiliations:** Department of Biology, McGill University, 1205 Docteur Penfield, Montréal, Quebec, Canada H3A 1B1

**Keywords:** competitive exclusion, fitness inequality, demographic stochasticity, resource competition

## Abstract

Traditional niche theory predicts that when species compete for one limiting resource in simple ecological settings the more fit competitor should exclude the less fit competitor. Since the advent of neutral theory ecologists have increasingly become interested both in how the magnitude of fitness inequality between competitors and stochasticity may affect this prediction. We used numerical simulations to investigate the outcome of two-species resource competition along gradients of fitness inequality (inequality in R*) and initial population size in the presence of demographic stochasticity. We found that the deterministic prediction of more fit competitors excluding less fit competitors was often unobserved when fitness inequalities were low or stochasticity was strong, and unexpected outcomes such as dominance by the less fit competitor, long-term co-persistence of both competitors or the extinction of both competitors could be common. By examining the interaction between fitness inequality and stochasticity our results mark the range of parameter space in which the predictions of niche theory break down most severely, and suggest that questions about whether competitive dynamics are driven by neutral or niche processes may be locally contingent.

## 1. Introduction

The competitive exclusion principle, which holds that only one species can persist per limiting resource or niche [1], is a long standing tenet of community ecology with both theoretical[2–5] and empirical support [6–8]. It positions competition as a major force in community dynamics with direct consequences for community composition, and consequently for biodiversity and ecosystem function.

Community ecology has traditionally explained competitive exclusion as the deterministic result of different competitive abilities among competitors, with the understanding that the most fit competitor should exclude other competitors unless differences exist between competitors that focus intraspecific competition relative to interspecific competition (i.e. niche differentiation) [5]. Recently, neutral theory [9,10] has emphasized the ecological importance of long term but unstable coexistence, and has challenged community ecologists to demonstrate that extinctions in competitive systems are indeed the result of competitive inequalities between species (e.g. [11]), and not simply the product of stochastic processes [10,12].

Universal neutrality in competitive communities is unlikely, but the question of how frequently competitive interactions are settled by fitness inequalities, as opposed to stochastic extinctions, remains open. Population geneticists have extensively examined how drift and selection affect allele frequencies depending on the strength of selection and the effective population size [13–15], and the same principles should presumably hold for species-level competitive dynamics [16]: in situations where fitness inequalities are strong, competitive outcomes are likely to follow deterministic predictions, but where inequalities are weaker the importance of stochasticity will probably be greater.

Fitness inequality, the relative competitive abilities of competitors in a given niche [17], defines the deterministic prediction for the outcome of competition in that niche. Resource competition theory [18] offers a useful framework with which to address fitness inequality. The theoretical simplicity of the resource competition framework is captured in a single value per resource, R*, which reflects the resource concentration at which a consumer-resource system will equilibrate when that resource is limiting and constitutes a measure of fitness in equilibrium [5] which predicts dominance by the competitor with the lowest R* value. While resource competition theory has typically been investigated in a deterministic context (e.g. [19,20]), stochastic extensions have also been studied (e.g. [21–23]).

Stochasticity, which represents the influence of random or unconsidered forces on population dynamics, can manifest as unpredictable variation in the fertility and mortality of individuals (demographic stochasticity), or as the effects of unpredictable environmental variation on all the individuals in a population (environmental stochasticity) [12]. While stochasticity can provide a basis for mechanisms of coexistence [5,23,24], it can also reduce diversity when it causes rare types to disappear from a community.

Both fitness inequality and demographic stochasticity affect the predictability of resource competition outcomes, but a full and systematic investigation of how fitness inequality and initial population size interact to affect competitive dynamics remains to be carried out. It is often unclear whether specific extinctions are due to deterministic or stochastic processes, or the combination of both, and an approach that systematically considers the influence of stochasticity along a broad range of fitness inequality values is essential in determining the breadth of conditions in which the predictions of niche theory are least reliable. In conditions where one competitor is markedly more fit outcomes are less likely to be affected by stochasticity than in cases where competitors are more evenly matched. While demographic stochasticity by definition strikes independently of fitness, large disparities in fitness are likely to quickly translate into large disparities in abundances, leaving the less fit competitor more exposed to both deterministic and stochastic extinction. Conversely, when fitnesses are relatively evenly matched, the community moves towards deterministic extinction more slowly, and both populations remain more or less equally vulnerable to demographic extinction for some time. Consequently, both fitness inequality and demographic stochasticity will affect how often the deterministic prediction of dominance by the more fit competitor is realized.

Here we model two-species competition for a single resource using a classical resource competition framework [8] to investigate the effects of varying fitness inequality and stochasticity on the outcome of competition across a range of population sizes and growth rates. We use R* as our metric of fitness and predict that under certain conditions the general deterministic prediction of complete dominance by the most fit competitor will often be violated:

(i) as fitness inequality weakens and initial growth rates are reduced we expect the timescale of persistence for both competitors will lengthen, potentially leading to co-persistence of both competitors. Both of these factors are likely to influence the amount of time needed for a consumer-resource system to achieve an equilibrium, and while resource competition theory suggests that competitive exclusion is inevitable in all but the most unrealistic of cases (i.e. a deterministic system with absolutely no fitness inequality), the magnitude of fitness inequality still plays an important role by determining the duration of co-persistence;
(ii) as initial population sizes become smaller and initial growth rates become lower, we expect that demographic stochasticity will become an increasingly prominent factor in determining the outcome of competition, increasing the probability that either one, or both species, will go extinct, even if not resource limited; and
(iii) as the strength of fitness inequality weakens or the strength of demographic stochasticity increases there will be increasing uncertainty in the identity of the species that dominates, with increased potential for the more fit species to go extinct stochastically and cede the habitat to dominance by the less fit species.

Recent work on the factors governing competitive outcomes have emphasized the simultaneous roles of fitness inequality and niche differentiation [5,17,25,26], but the consequences of variable fitness inequality when niche differentiation is absent have been relatively unexplored. Consequently, we have restricted our attention to systems solely governed by fitness inequality, and we demonstrate that when the strength of stochasticity is great, or when population dynamics are slow, knowledge of fitness inequalities may not be enough to predict competitive outcomes.

## 2. Material and methods

### 2.1 The model

We used a discretized version of a classical resource competition framework [8] based on Michaelis–Menten (Monod) dynamics to simulate resource competition between two species for a single mineral resource in an open system:

2.1 $$\begin{array}{rl} N_{i}(t+1) & =N_{i}(t)+\frac{\mu_{i}N_{i}(t)R(t)}{K_{i}+R(t)}-DN_{i}(t), \end{array}$$

2.2 $$\begin{array}{rl} R(t+1) & =R(t)+D(I-R(t))-\sum_{i}\frac{Q_{i}\mu_{i}N_{i}(t)R(t)}{K_{i}+R(t)}, \end{array}$$

2.3 $$\begin{array}{rl} \text{which yields }R_{i}^{*} & =\frac{DK_{i}}{\left(\mu_{i}-D\right)}, \end{array}$$

where *N*_*i*_(*t*) is the density of species *i* at time *t*; *R*(*t*), the concentration of the resource at time *t*; *μ*_*i*_, the maximum growth rate of species *i*; *K*_*i*_, the half-saturation constant of species *i*; *D*, the dilution rate; *I*, the concentration at which new resources inflow; *Q*_*i*_, the amount of resource taken up by new individuals of species *i*; and $R_{i}^{*}$, the equilibrial resource concentration of a system dominated by species *i*.

To investigate the effects of fitness inequality we explored competition at 35 points in a parameter space in which *μ*_*i*_ varied so that each species' R* value increased linearly from one extreme of the parameter space to the other, with the same slope, but in opposite directions (electronic supplementary material, figure S1). Both species R* values were identical at the central point in parameter space (point 18), where fitness inequality was 0. We defined relative fitness as the R* value of one competitor divided by the R* value of the other for any given point in the parameter space, and we defined fitness inequality as the absolute difference in the relative fitnesses of the two competitors at a given point in this parameter space.

While we ultimately see our model as offering a general demonstration of the consequences of fitness inequality and demographic stochasticity, we chose parameter values within the range of a realistic phytoplankton system (table 1), and describe the model here using the units relevant to such a system. To investigate how our model would perform under relatively low and high growth rates, we let *μ* vary over two different ranges of values. In the low growth rate case, *μ* had a median value of 0.105 d^−1^ (barely greater than the dilution rate), whereas in the high growth rate case, *μ* had a median value of 0.4 d^−1^. One of the consequences of modelling two ranges of *μ* with preset R* values is that to investigate these two ranges, while retaining the same R* values, required that the value of *K* that we used change, and in the same direction as *μ*. Consequently, in our low growth model *K* was constant at 0.072 μmol l^−1^, and in the high growth model *K* was constant at 4.32 μmol l^−1^. As a result, one set of models had both high *μ* values and a high value for *K*, and the other set had low *μ* values and a low value for *K*. *Per capita* growth rates, *μ*_*i*_*R*(*t*)/(*K*_*i*_+*R*(*t*)), are more sensitive to changes in *μ* when resources are abundant (i.e. early in our simulations), and consequently initial growth will be relatively rapid when *μ* and *K* take on high values, and slower when both *μ* and *K* take on lower values, even though both combinations were constrained to give the same equilibrial fitness. For simplicity, we term the models looking at the two ranges in *μ* ‘low-growth’, and ‘high-growth’ for the duration of the paper, though it is worth remembering that when resources are low the relative growth rates of our models reverse (electronic supplementary material, figure S2). Dilution of the system (*D*), which represents the rate of inflow and outflow of materials (i.e. resources and consumers), was held constant at 0.1 d^−1^, though to assess the robustness of our findings we also ran simulations for another set of dilution rates (see below). We arbitrarily parametrized *Q* to have a value of 1×10^−6^ μmol individual^−1^ for both competitors, and resource inflow at 10 μmol l^−1^, which was also chosen as the resource concentration at the beginning of the simulations.

**Table 1.** Representation of the parameters and variables in the model, the parametrizations we chose, examples of units for which these parametrizations could be realistic for a phytoplankton system and supporting references to empirical work (either experimental or field research) for these parametrizations.

parameter or variable name	nature	units for which our model could realistically model a phytoplankton system	range or value of parameter	supporting reference for our parametrizations
*N*	population size	individuals l^−1^		
*R*	concentration	μmol l^−1^		
*μ*	rate	day^−1^	0.104–0.107 (low growth); 0.343–0.493 (high growth)	[11,18,27,28]
*K*	concentration	μmol l^−1^	0.072 (low growth); 4.32 (high growth)	[8]
*D*	rate	day^−1^	0.1	[27]
*I*	concentration	μmol l^−1^	10	[29]
*Q*	concentration⋅individual^−1^	μmol individual^−1^	1×10^−6^	[8]
R*	concentration	μmol l^−1^	1.10–1.78	[8]

To introduce the effects of stochasticity to the traditional resource competition framework of equations (2.1) and (2.2), we allowed the population size in the next time step to be the sum of the population size in the previous time step, a Poisson draw for growth, and a modified Poisson draw for death (modified to prevent stochastic mortality from surpassing the current population size), with the expectations of the Poisson draws being the deterministic predictions for these two terms [30,31] based on values at time *t*, or more formally

2.4 $$\begin{array}{rl} N_{i}(t+1) & =N_{i}(t)+\mathrm{pois}(G)-\mathrm{minimum}(\mathrm{pois}(M),N_{i}(t)), \end{array}$$

2.5 $$\begin{array}{rl} \text{where }G & =\frac{\mu_{i}N_{i}(t)R(t)}{K_{i}+R(t)} \end{array}$$

2.6 $$\begin{array}{rl} \;\text{and}\;M & =N_{i}(t)D. \end{array}$$

No stochasticity was applied directly to the resource equation, though the resource concentration experienced indirect stochasticity as we forced resource uptake by the consumer to be equal to the actual growth that each competitor achieved, while verifying that the required resource uptake did not exceed the available resources.

Populations were deemed to have gone extinct once *N*_*i*_(*t*+1)<1, and cases where both competitors had gone extinct by the end of simulations were termed dual extinctions. Cases where neither competitor had gone extinct by the end of the simulations were termed co-persistence, and the cases where only one competitor had gone extinct were termed less fit dominant, more fit dominant or dominance by one species at fitness equality, depending on the fitness inequality that characterized the simulated competition.

Populations were seeded with equal numbers of both competitors at one of 10 levels of initial population size (1, 2, 4, 8, 16, 32, 64, 128, 256 or 512 individuals). These initial population sizes formed the stochasticity treatment for our stochastic model, as the form of stochasticity was held constant (i.e. a Poisson distribution). Each population×environment treatment was replicated 100 times, and simulations were run for 20 000 time steps. All simulations were conducted in Python v. 3.3.3 using the numpy package v. 1.8.0, and exported to R for analysis using the rpy2 package v. 2.3.8. Analyses of resulting data were done in R v. 3.0.2 [32]. Contour plots were made using the filled.contour3 and filled.legend functions (https://r-forge.r-project.org/scm/viewvc.php/pkg/R/filled.contour3.R?view=log&root=detsel; http://wiki.cbr.washington.edu/qerm/sites/qerm/images/2/25/Filled.legend.R). The script files needed to execute and analyse the model are publicly available (see ‘Data accessibility’ section).

### 2.2 Assessing generality

To assess the generality of our model, we ran a number of additional simulations (electronic supplementary material, table S1) which are briefly described below, and the results of which are found in the electronic supplementary material. To compare our stochastic results to a deterministic baseline, we executed a model with a deterministic framework based solely on equations (2.1) and (2.2). Whereas *N*_*i*_ was a discrete variable in our stochastic framework, in the deterministic case population size was continuous (with an extinction threshold at *N*_*i*_<1) as simulations with small initial population sizes yielded population growth rates too low for any growth when population sizes were conservatively constrained to integer values. To assess the generality of our findings we also investigated deterministic and stochastic frameworks that varied *K* with R* instead of *μ* (while holding *μ* constant at the median values it took in the main models). We also ran our model with alternative dilution rates of 0.01 d^−1^ and 0.05 d^−1^ (while varying either *μ* or *K*). We parametrized the alternative dilution rate models by retaining the R* values, along with the constant parameter (either *μ* or *K*) from the model where dilution was set at 0.1 d^−1^ (i.e. 0.105 d^−1^ or 0.4 d^−1^ in the case of *μ*; 0.072 μmol l^−1^ or 4.32 μmol l^−1^ in the case of *K*), and recalculated the varying parameter using these constants and the appropriate dilution rate (electronic supplementary material, figure S3).

## 3. Results

Our simulations showed that while the predictions of resource competition theory were often borne out in stochastic systems, certain conditions defined by fitness inequality, initial population size and growth rate resulted in different outcomes. While these factors clearly interact, it is still possible to analyse their effects individually, and we describe these effects while highlighting interesting interactions.

### 3.1 Effects of fitness inequality

**Figure 1.**
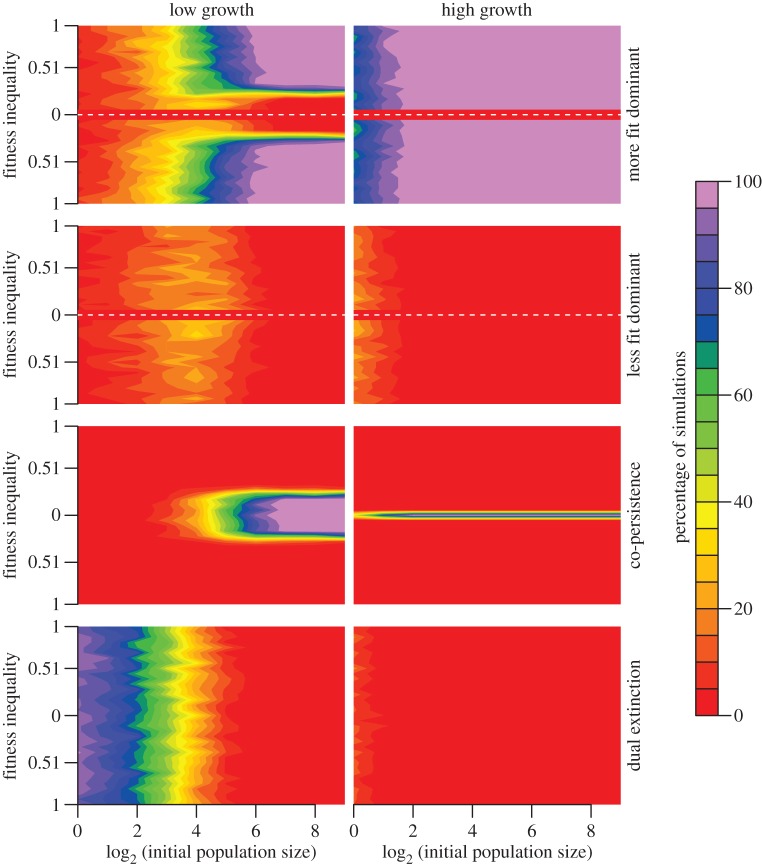
The percentage of simulations yielding four of five possible outcomes as a function of initial population size, fitness inequality (along the 35 points in parameter space) and growth rate. Results from the low growth model are in the left column, and results from the high growth model are in the right column. Each row represents a different potential result outcome: the first row shows simulations in which the more fit competitor has dominated, the second row shows simulations in which the less fit competitor has dominated, the third row gives the percentage of simulations in which both competitors have persisted to the end of simulations and the fourth row shows simulations in which neither competitor has persisted to the end of simulations. Note that by definition the first and second rows have values of 0 when competitors have equal fitness because neither competitor can be more or less fit when there is no fitness inequality.

The outcome of resource competition varied widely across the fitness inequality gradient (figure 1). As the two competitors experienced increasing fitness inequality there was a marked trend towards greater dominance of the more fit competitor, especially for larger initial population sizes and in the model with high growth rates. In the model with high growth rates, the transition from equality (and thus either co-persistence, dominance by one competitor at equality or dual extinction) to complete dominance by the more fit competitor was rapid (figure 1, row 1–column 2), effectively taking place as soon as the environment left fitness equality (i.e. 0.056 fitness inequality). Conversely, in the low growth rate model the transition to complete dominance by the more fit competitor was not complete until fitness inequalities were stronger (i.e. 0.450 fitness inequality) (figure 1, row 1–column 1). There was also a trend towards greater dominance of the less fit competitor as fitness inequality weakened, though in general, dominance by the less fit competitor was less common (ranging from 0% to 34% of simulations, depending on the treatment), and thus the trend was less pronounced (electronic supplementary material, figure S4).

The co-persistence of both competitors until the end of the simulations was highly contingent on the relative fitness of the competitors, with the frequency of co-persistence decreasing as the fitness inequality grew (figure 1, row 3). While this effect was observed in both low and high growth models, the extent to which it was realized was clearly dependent on growth rates, co-persistence being found as far as 0.393 fitness inequality in the low growth model, but was only found at equality in the high growth case. By contrast, dual extinction of competitors was largely unaffected by fitness inequality (figure 1, row 4).

### 3.2 Effects of initial population size

**Figure 2.**
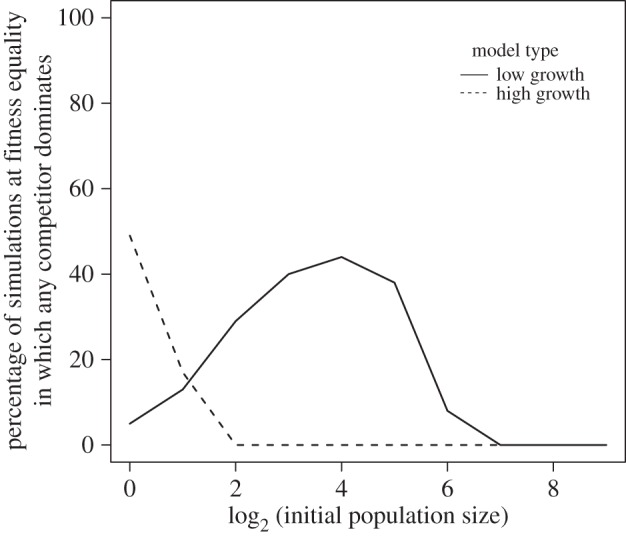
The percentage of simulations at fitness equality (parameter space value 18) that yield the dominance of one competitor (either species) as a function of initial population size and growth rate.

Initial population size affected the probability of observing all the potential competitive outcomes. In general, dominance by the more fit competitor and co-persistence increased as the initial population size increased (figure 1, rows 1,3), while cases of dual extinction decreased as initial population size increased (figure 1, row 4). The frequency of dominance of the less fit competitor (figure 1, row 2), and the dominance of either competitor at fitness equality (figure 2) were also affected by initial population size, but the relationship between population size and frequency was dependent on growth rates: in the low growth model both had unimodal relationships to initial population size with maxima at intermediate population sizes, whereas at high growth rates the frequency of both declined as initial population size increased.

### 3.3 Variation in growth rates

There were clear effects of growth rates on the outcome of resource competition (figure 1). In the high growth rate model, the more fit competitor more often came to dominance, effectively dominating all communities where initial population sizes were sufficient to prevent stochastic extinction (figure 1, row 1), whereas in the slower growth model, universal dominance by the more fit competitor was confined to higher fitness inequalities and initial population sizes (e.g. in the low growth rate model complete dominance by the more fit competitor was only reliably achieved at the three highest initial population sizes when fitness inequalities were maximal; conversely, in the high growth rate model dominance was reliably achieved at all but the three lowest initial population sizes). Higher growth rates also effectively prevented stochastic extinction at smaller population sizes than in slow growth models (figure 1, row 4), and reduced the frequency with which the less fit competitor dominated (figure 1, row 2; electronic supplementary material, figure S4). Dominance by one competitor when fitness was equal was also affected, with the high growth rate model achieving maximum dominance at lower population sizes than the low growth rate model (figure 2). The effects of initial growth rates on co-persistence were more subtle, as higher growth rates led to an increase in the proportion of simulations yielding co-persistence near fitness-equality (i.e. co-persistence remained common even at lower initial population sizes), but a reduction in the proportion of simulations yielding co-persistence when fitness inequalities were greater (figure 1, row 3).

## 4. Discussion

Recent debate has focused on whether natural communities are niche structured or neutral [10,17,33,34], and by extension, whether the outcomes of competition in nature are predicted by fitness differences. Our results demonstrate that, even when assuming a resource competition framework, the predictability of competitive outcomes depended on all three of the factors we varied: the magnitude of fitness inequality, the strength of demographic stochasticity and the magnitude of growth rates that competitors experience. We show that the prediction from niche-theory of dominance by the most fit competitor is most often realized when fitness inequalities are great, initial population sizes are high (and thus demographic stochasticity is weak) and growth rates are high. When any of these conditions are not met it becomes increasingly likely that an outcome not predicted by resource competition theory (i.e. dominance of the less fit competitor, extinctions of both competitors, or the persistence of both competitors) will be realized, and the likelihood of each outcome is defined by the interaction of fitness inequality and initial population size. Our model thus suggests that to answer questions about how ecological communities are structured ecologists will need to understand: (i) the magnitudes of fitness inequalities between competitors in the relevant environment, (ii) how prevalent stochasticity is in that community, and (iii) at what pace ecological interactions take place. We find the first question particularly interesting, as empirical evidence shows fitnesses vary along environmental gradients and suggests that regions of effective equivalence may exist [27,35,36]. Consequently, competitive systems may simultaneously behave in niche or neutral structured fashions depending on where a community finds itself along an environmental gradient, and how stochasticity and dynamics play out in that community. Ultimately, our results suggest that the potential for neutral dynamics in ecological communities may be extremely locally contingent, and while approaches that discuss fitness inequality offer unique insights to this problem, fitness inequality *per se* is not sufficient to make completely reliable predictions about how communities will be structured [17].

Our model makes several assumptions that affect the extent to which our findings are likely to be realized in empirical systems. The resource competition model we used has been well tested in both laboratory and field [8,37,38], and makes clear predictions about competitive outcomes at equilibrium when competition is for a single limiting resource. The competitors in our simulations competed for a single limiting resource, but our use of a stochastic model, and our choice to model in discrete time with a limited number of time steps, does mean that our co-persistence results may include simulations where the expected outcome will eventually manifest, but has not done so by the end of our simulations (indeed, our deterministic simulations frequently resulted in the co-persistence of competitors; electronic supplementary material, figure S5). Still, the fitness inequality defined by the competitors' R* values was an excellent predictor of competitive outcome in conditions where stochasticity was minimal and the pace of competition was quick. Where these conditions were not met we found exceptions to the predictions of R* theory, such as regions of competitive co-persistence around fitness inequality, and the dominance of the less fit competitor. Furthermore, we assumed a non-spatial model of competition, and it is possible that spatial systems might experience either reduced departures from the deterministic prediction owing to mitigating immigration or exacerbated departures from the deterministic prediction owing to priority effects [21].

The relevance of our quantitative results to natural communities will depend on how closely our parameter values and implementation of stochasticity reflect those found in these communities. With this obvious cautionary note in mind, we chose parameter values that could be realistic for some organisms and resources at a given timescale (e.g. phytoplankton on a daily timescale), and the consistency of the qualitative patterns in our results across a variety of parameter values suggests that the patterns we describe are likely to occur in natural systems. We assumed that demographic stochasticity can be described by Poisson variance in both birth and death processes, a common assumption in ecology and population genetics [30,31,39]. We chose to manipulate the strength of demographic stochasticity by varying initial population size, and while this is a reasonable method for manipulating stochasticity, it is worth noting that simulations which started with larger population sizes will have initially exerted stronger effects on the resource abundance and thus progressed more quickly towards deterministic exclusion in addition to having lowered stochasticity.

The key qualitative patterns of our model are reasonably robust to variations in the parameters used to model growth, and the parameter by which R* was varied. Variation in the dilution rate did in some cases lead to results that were different from our main model, but that do not challenge our conclusions (electronic supplementary material, figures S5–S8). Obviously, though we feel the assumptions of our model are justifiable, they are also simplistic. While some natural systems may be perennially limited by the same resource, others probably deal with a variety of challenges such as predation, disease or new resources becoming limiting. Here we wished to investigate the effects of fitness inequality in a situation where it could be relatively easily quantified, but natural competitive systems may be more complicated.

A number of questions present themselves for future research. Our model implements only demographic stochasticity and not environmental stochasticity. The effects of demographic stochasticity are strongest at small population sizes, whereas the effects of environmental stochasticity are largely independent of population size [12], and thus it is likely that introducing environmental stochasticity will increase the risk of extinction even in well-established populations [22,40]. Consequently, the combination of demographic and environmental stochasticity might further confound the influence of fitness inequality on the outcome of competition [30]. Alternatively, environmental stochasticity might reinforce the effects of fitness inequality if less fit competitors are also more strongly affected by environmental stochasticity. Empirically, our model begs the question about how competitive fitness varies along environmental gradients in space and time, and how these environment-fitness gradients map onto real world settings [41]. Should regions of relative equality prove to be common in natural communities, particularly those in which competition occurs slowly or in which stochasticity is strong, it may well be that the predictability of competitive interactions cannot be taken for granted. Our results predict that co-persistence of competitors, or dominance by less fit competitors may be surprisingly common, and it would be exciting to see if this prediction holds in the field.

## 5. Conclusion

The ability to predict outcomes is a virtue for any scientific theory, and niche theory in particular makes strong predictions about when species should dominate ecological communities. Recent debate about the extent to which competitive outcomes can be successfully predicted has sparked questions about the suitability of specific competitive frameworks [42–44], yet our results demonstrate that even within a single niche framework there can be considerable uncertainty in competitive outcomes if fitness inequalities are modest, growth rates are slow or demographic stochasticity is strong. When any of these conditions are met unexpected outcomes, such as co-persistence, or dominance by the less fit competitor may emerge. Thus, the extent to which competitive outcomes will be predictable in natural communities will depend on the environmental conditions present, and how these relate to the competitors' experience of differences in fitness and stochasticity in their environment.

## Supplementary Material

Online Supplementary Materials: Results, and discussion of analyses of generality of our model, supplementary figures and table.

## Data accessibility

The data and scripts supporting this article are available at the Dryad digital repository (http://dx.doi.org/10.5061/dryad.16n10).
