# Supplementary material for: Predicting the outcome of competition when fitness inequality is variable
Source: R Soc Open Sci. 2015 Aug 12;2(8):150274. doi: 10.1098/rsos.150274 (PMC4555862; doi:10.1098/rsos.150274)
Supplement: Online Supplementary Materials: Results, and discussion of analyses of generality of our model, supplementary figures and table. [file rsos150274supp1.pdf]

Online supplementary materials for Pedruski et al. *Predicting the outcome of competition when fitness inequality is variable*

Please refer to Table 1 for relevant units for all parameters and variables.

1 Results of models assessing generality

1.1 Effect of stochasticity

Competitive outcome was strongly affected by fitness inequality and growth rate in the deterministic version of our model, but was not affected by initial population size (Fig. S5). As with the stochastic model, dominance by the more fit competitor was restricted to simulations with some amount of fitness inequality, and co-persistence was restricted to simulations with relatively little fitness inequality between the competitors, though the fitness inequalities that resulted in co-persistence were more constrained to fitness equality in the deterministic model in the low growth conditions (similarly, the fitness inequalities resulting in dominance by the more fit competitor were more constrained in the deterministic low growth model). The deterministic model resulted in no dominance by the less fit competitor, dominance by any competitor at fitness equality, nor any dual extinction.

1.2 Effect of varying dilutions

Within both our deterministic and stochastic model frameworks there was a clear effect of varying the dilution rate on the outcome of competition realized at 20000 time steps, with increasing dominance by the more fit competitor as dilution increased, and decreasing co-persistence (Fig. S5, S6). In the stochastic model, where other results were possible, dilution had no discernible effect on the amount of dominance by any competitor at fitness equality, and any effects on the amount of dominance by the less fit competitor, or the amount of dual extinction, were relatively weak. Generally, varying dilution rate had a greater effect on competitive outcome in low growth models than in the high growth models. The relationship between fitness inequality and the different potential outcomes of competition noted in the main (stochastic) model held except in the case of the lowest dilution rate in the low growth model where there was no obvious relationship between fitness inequality and dominance by either competitor, co-persistence, or dual extinction. In this case we expect the slow growth rate combined with the low dilution rate limited dynamics to such an extent that only demographic stochasticity had time to cause extinctions by the end of the simulations.

1.3 Effect of varying K (as opposed to μ)

The results of models varying K (Fig. S7, S8) were in many cases quite similar to the models where μ was varied (Fig. S5, S6), though there were a number of obvious differences. Whereas the μ-varying model returned no clear relationship between fitness inequality and co-persistence at the lowest dilution rate, all the models that varied K showed that co-persistence was found only in conditions of relatively minimal fitness inequality. Dual extinction was somewhat less common in K varying models than μ varying models where dilution rates were low. Finally, the relationships between dilution rate and the frequency of competitive outcomes differed in some ways from the relationships with μ varying models: in the low growth models there were clear effects of dilution rate on dominance by any competitor at fitness equality when K was varied (Fig. S9), and the monotonic relationships between dilution rate and both dominance by the more fit competitor and co-persistence were not held by the low growth deterministic model that varied K - in this case the fitness inequality conditions that resulted in co-persistence were most restricted at intermediate dilution rates.

2 Discussion of models assessing generality

Though some of the patterns observed in our focal model (stochastic, varying μ, D=0.1) were not represented in all of the models we deployed to assess generality we feel that none of these deviations challenge our conclusions. Many of these differences are trivial (e.g. the lack of any effect of varying initial population size in the deterministic model), and others most likely reflect different timescales of competition as opposed to any fundamental violation of our understanding of the focal model. For example, the broad co-persistence in the low growth models varying μ and having a dilution of 0.01 is likely due to a combination of low stochastic mortality and growth rates that do not vary strongly at low resource concentrations (the difference in net growth rate between highly fit and unfit individuals narrows in the μ-varying model as resource concentrations decline).

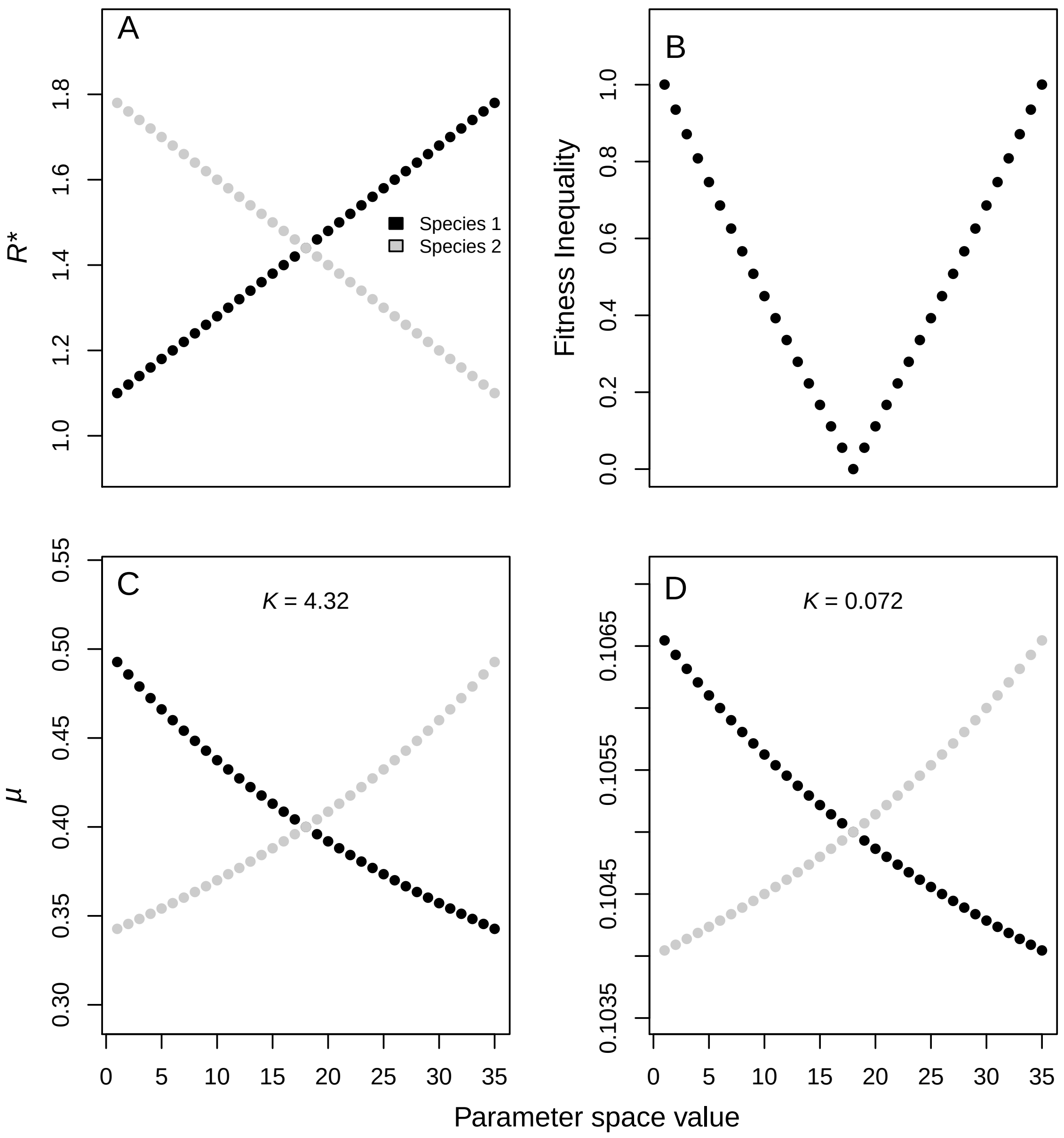

Figure S1: R^* values for both species in our focal model (stochastic, varying μ, D=0.1) through parameter space (A), the fitness inequality between the competitors through parameter space (B), and the μ values and K constants used to parameterize the low growth rate (C), and high growth rate models (D).

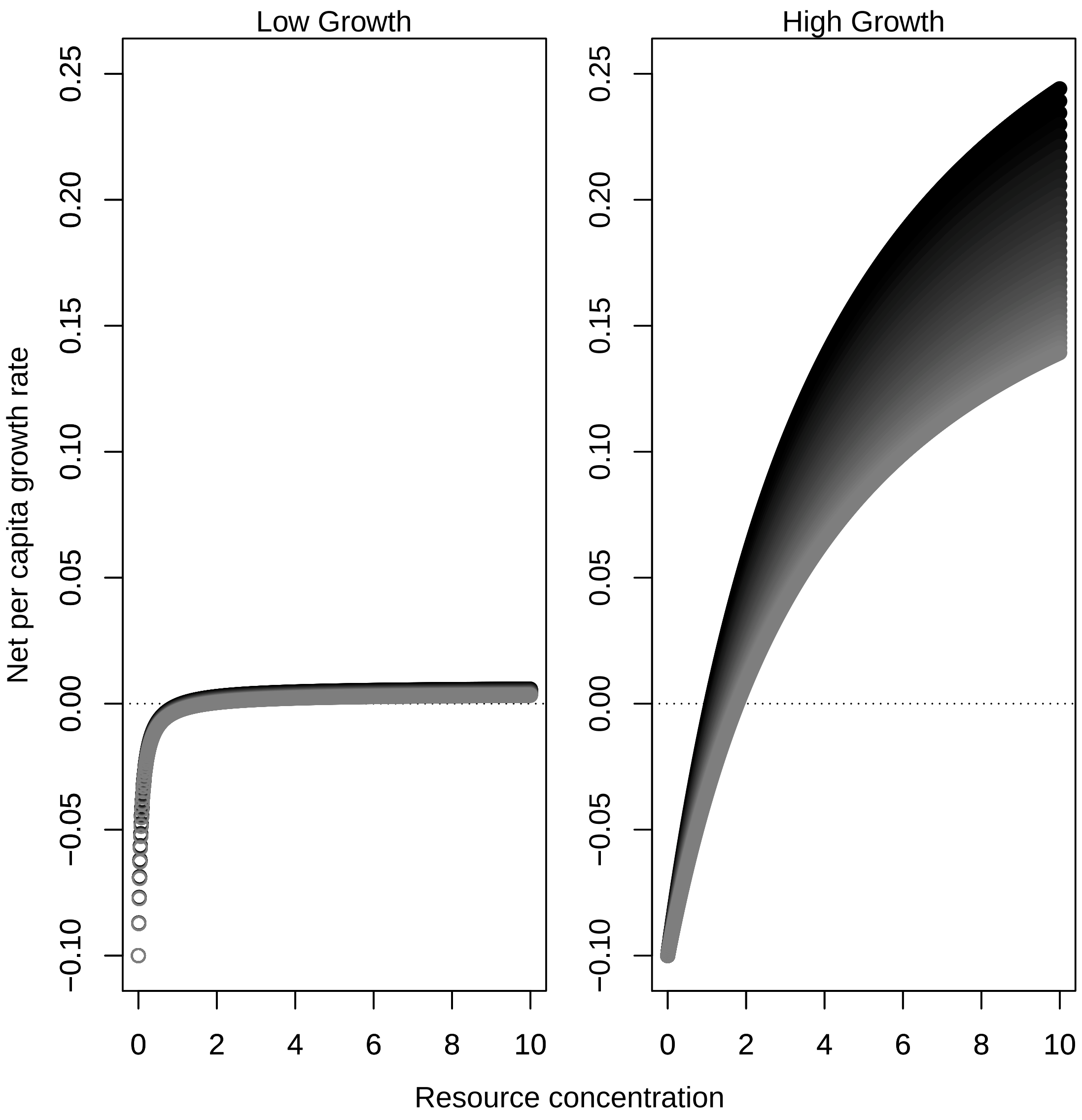

Figure S2: Deterministic net per captia growth rates as a function of resource concentration and the parameter space (darker greys represent parameter space values that give a fitness advantage) in the focal model. Dotted line indicates 0 growth, and therefore $R*$.

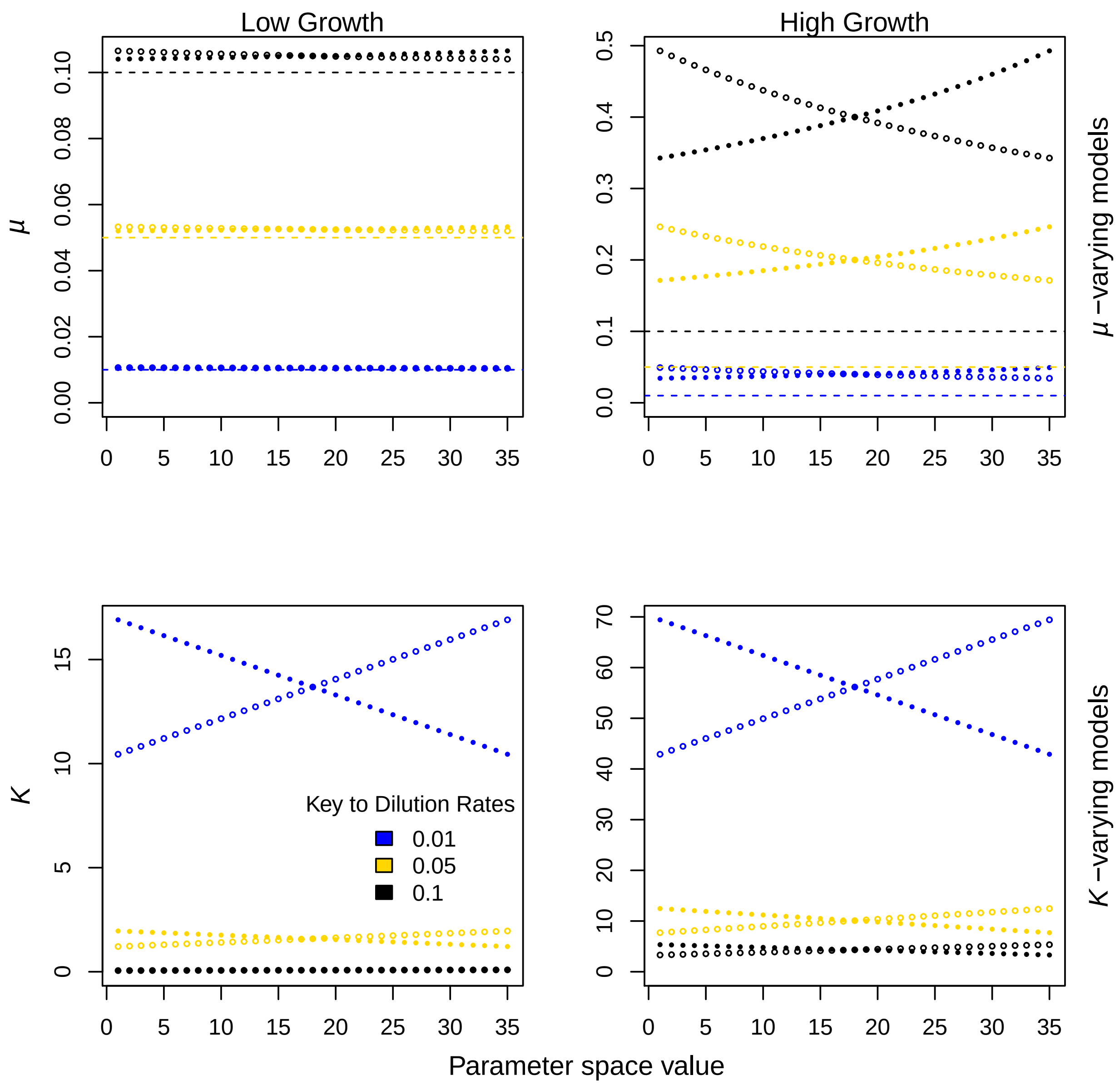

Figure S3: μ and K parameters used in simulations that varied dilution rate. Note that in the cases where μ varied (top row) K was either fixed at 0.072 (low growth) or 4.32 (high growth), and in the cases where K varied (bottom row) μ was either fixed at 0.105 (low growth) or 0.4 (high-growth). Species 1 values in filled points, species 2 values in open points, and dilution rates figured as dashed lines in μ-varying plots.

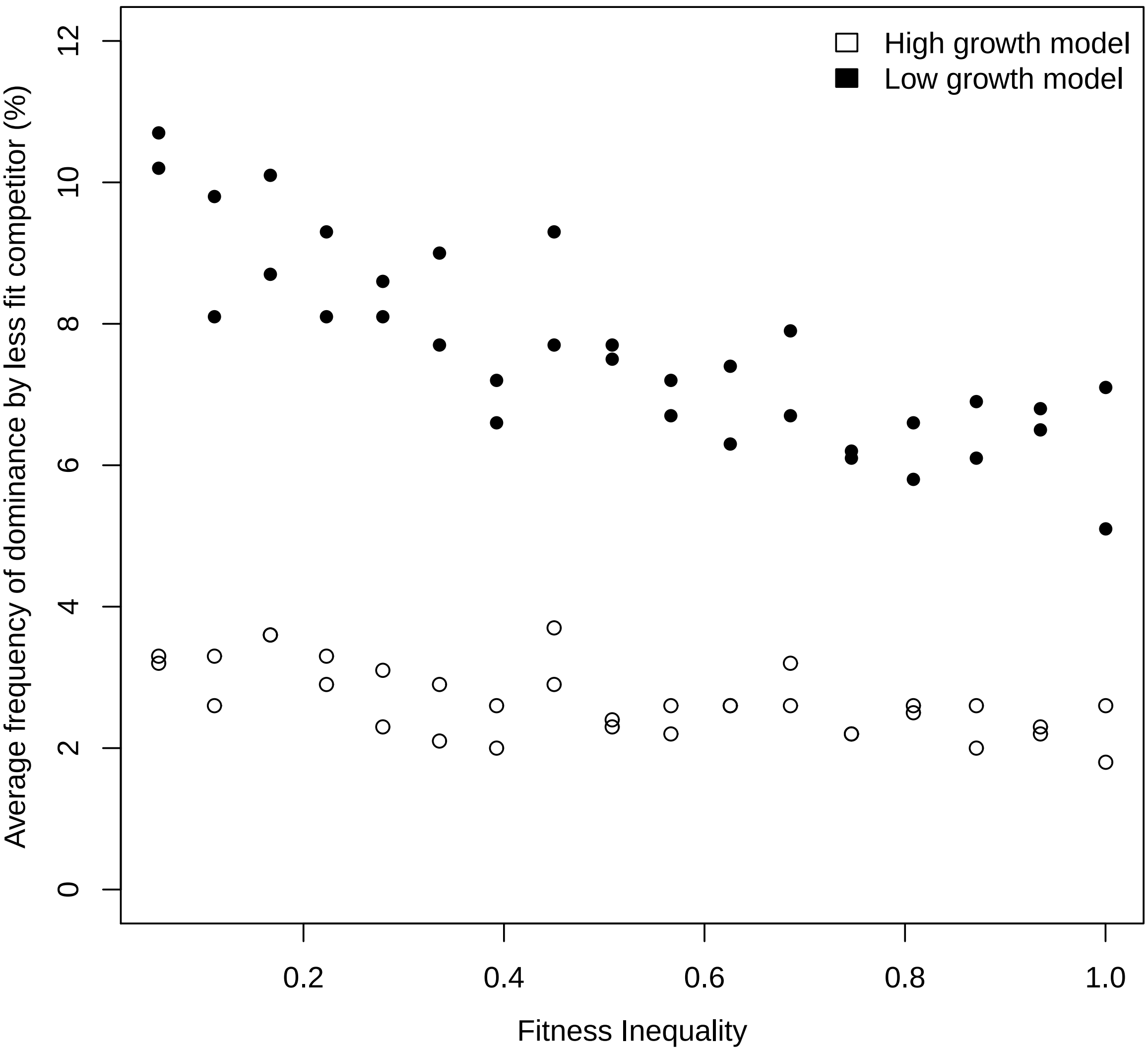

Figure S4: The percentage of simulations resulting in the dominance of the less fit competitor averaged across all initial population sizes as a function of fitness inequality and the relative growth rate in our focal model (stochastic, varying μ, D=0.1). The two results for each fitness inequality value (except 0 fitness inequality, where by definition dominance of the less fit competitor was impossible) reflect the use of the absolute difference in calculating fitness inequality across the 35 points in parameter space.

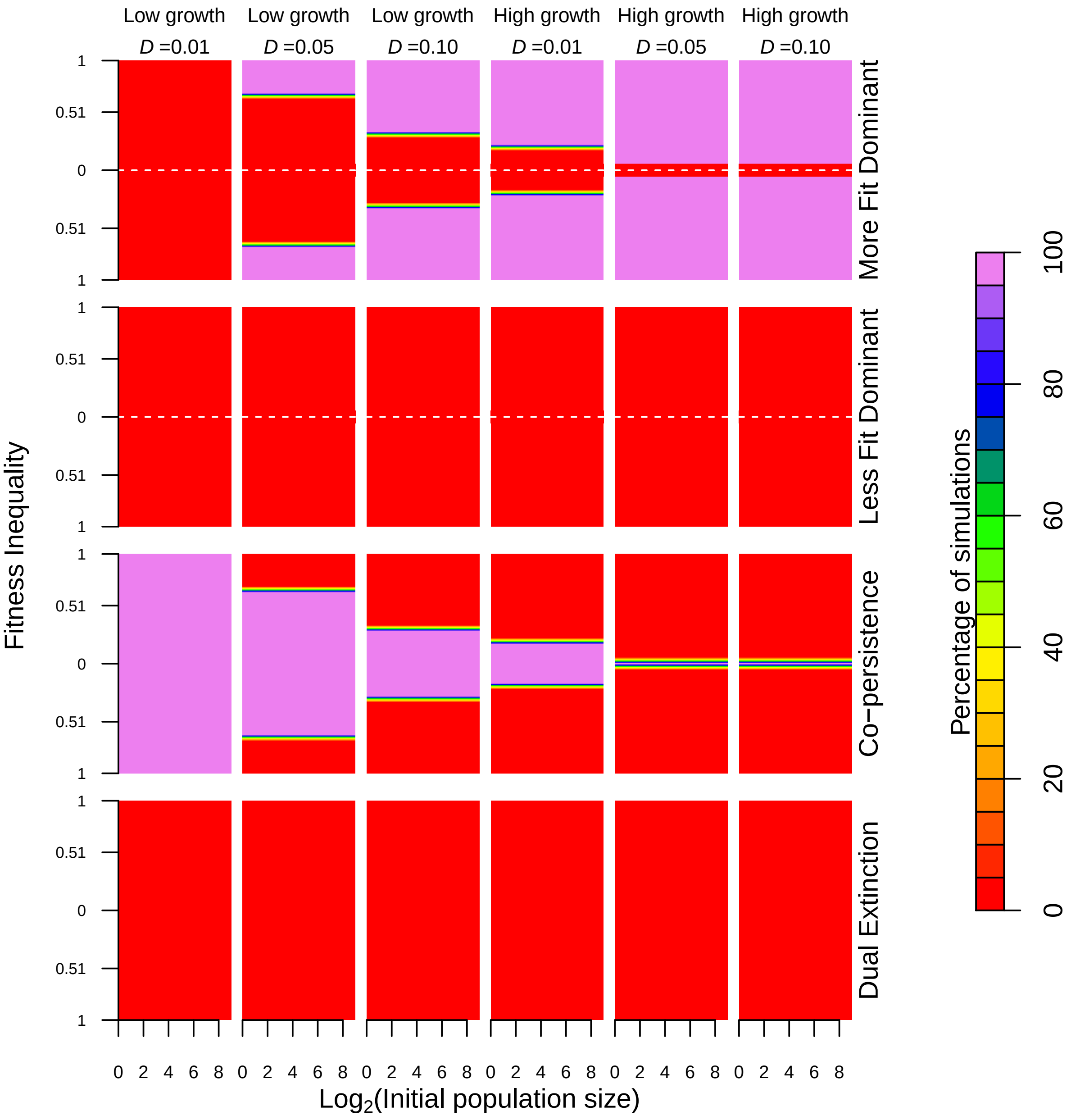

Figure S5: The percentage of simulations yielding four of five possible outcomes as a function of initial population size, fitness inequality (along the 35 points in parameter space), and growth rate when μ is varied in the deterministic framework at three different dilution rates. Each column represents simulations from a combination of dilution and growth rates, and each row represents a different potential result outcome: the first row shows simulations in which the more fit competitor has dominated, the second row shows simulations in which the less fit competitor has dominated, the third row gives the percentage of simulations in which both competitors have persisted to the end of simulations, and the fourth row shows simulations in which neither competitor has persisted to the end of simulations. Note that by definition the first and second rows have values of 0 when competitors have equal fitness because neither competitor can be more or less fit when there is no fitness inequality.

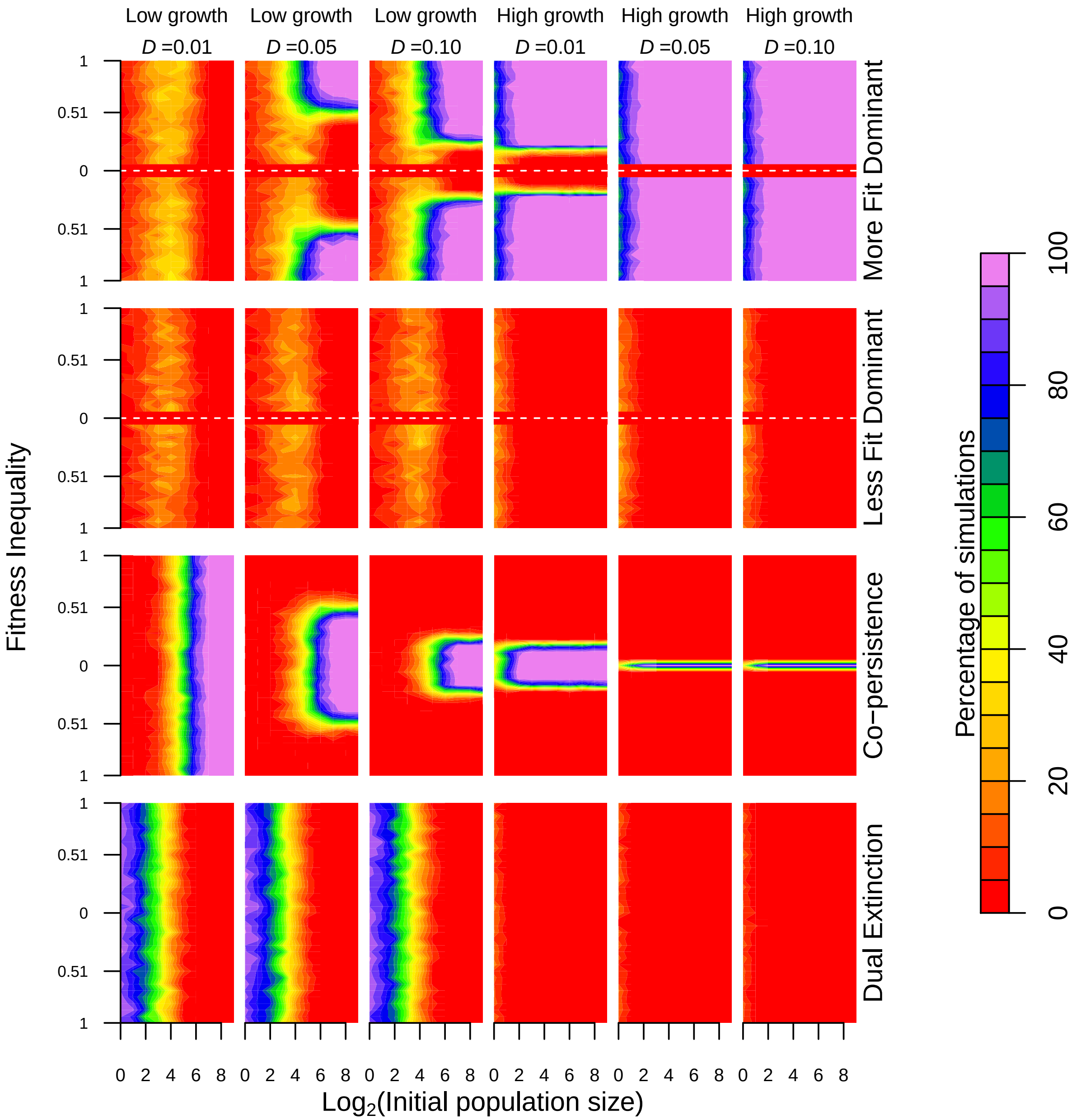

Figure S6: The percentage of simulations yielding four of five possible outcomes as a function of initial population size, fitness inequality (along the 35 points in parameter space), and growth rate when μ is varied in the stochastic framework at three different dilution rates. Each column represents simulations from a combination of dilution and growth rates, and each row represents a different potential result outcome: the first row shows simulations in which the more fit competitor has dominated, the second row shows simulations in which the less fit competitor has dominated, the third row gives the percentage of simulations in which both competitors have persisted to the end of simulations, and the fourth row shows simulations in which neither competitor has persisted to the end of simulations. Note that by definition the first and second rows have values of 0 when competitors have equal fitness because neither competitor can be more or less fit when there is no fitness inequality.

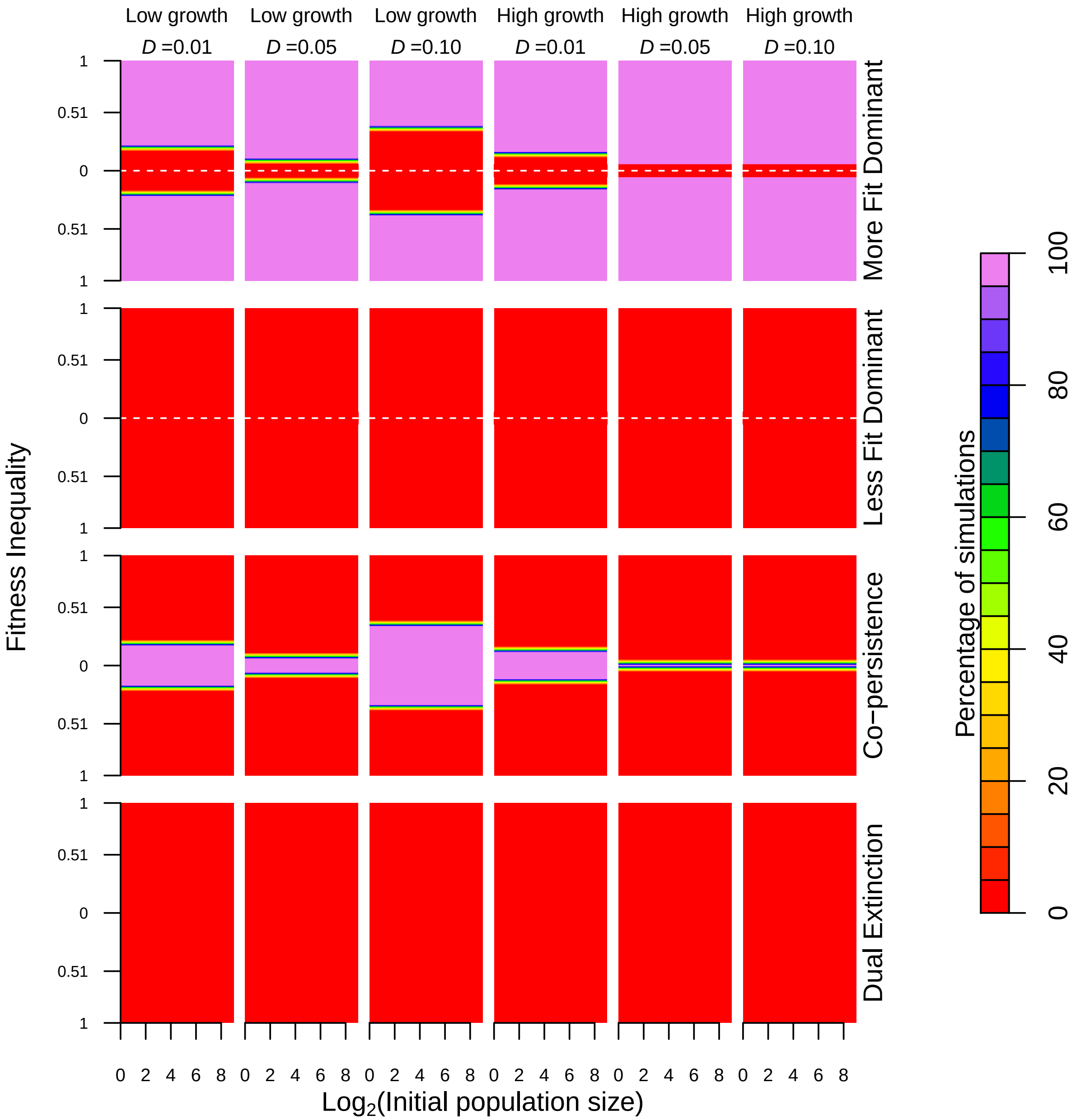

Figure S7: The percentage of simulations yielding four of five possible outcomes as a function of initial population size, fitness inequality (along the 35 points in parameter space), and growth rate when K is varied in the deterministic framework at three different dilution rates. Each column represents simulations from a combination of dilution and growth rates, and each row represents a different potential result outcome: the first row shows simulations in which the more fit competitor has dominated, the second row shows simulations in which the less fit competitor has dominated, the third row gives the percentage of simulations in which both competitors have persisted to the end of simulations, and the fourth row shows simulations in which neither competitor has persisted to the end of simulations. Note that by definition the first and second rows have values of 0 when competitors have equal fitness because neither competitor can be more or less fit when there is no fitness inequality.

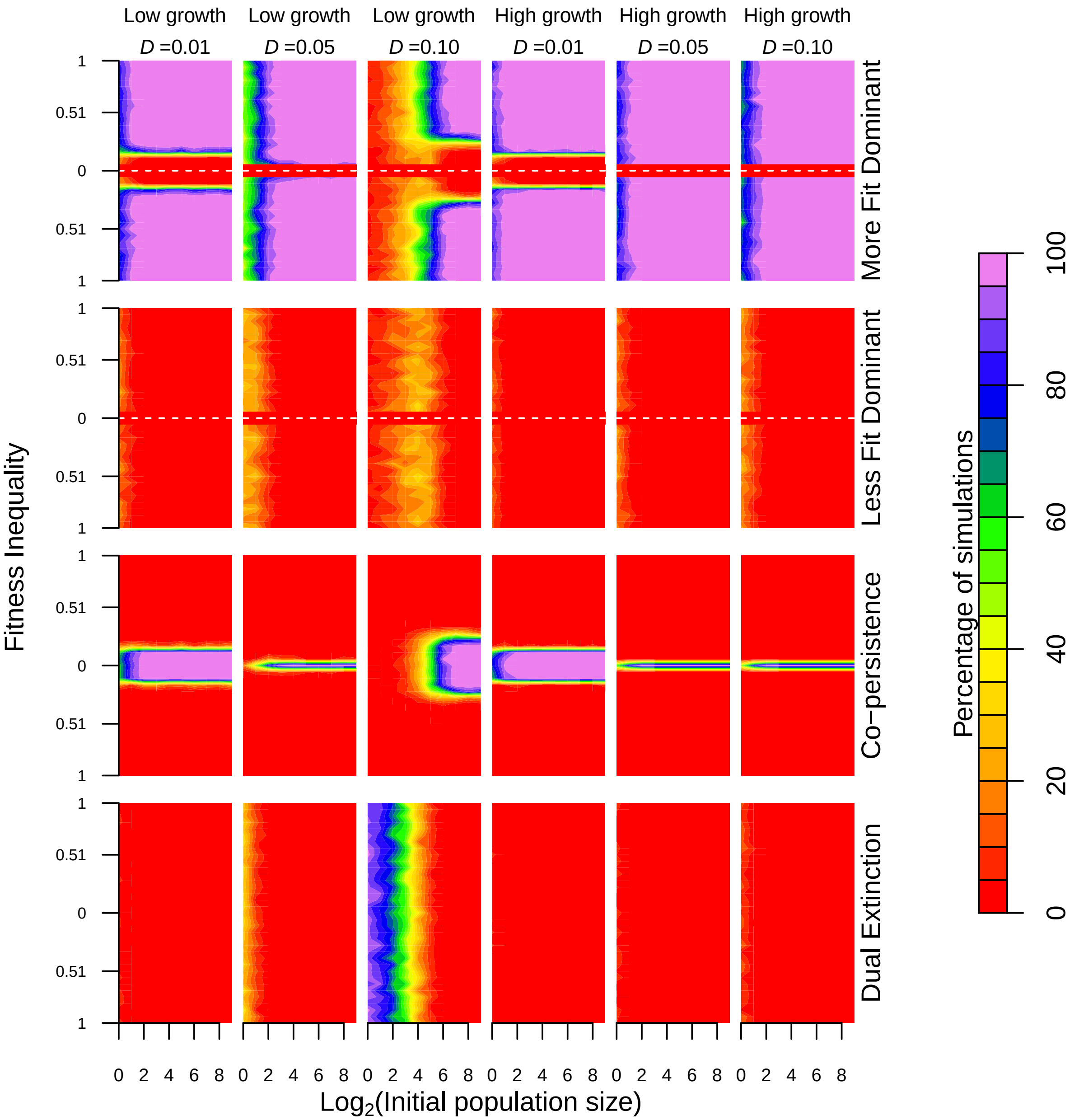

Figure S8: The percentage of simulations yielding four of five possible outcomes as a function of initial population size, fitness inequality (along the 35 points in parameter space), and growth rate when K is varied in the stochastic framework at three different dilution rates. Each column represents simulations from a combination of dilution and growth rates, and each row represents a different potential result outcome: the first row shows simulations in which the more fit competitor has dominated, the second row shows simulations in which the less fit competitor has dominated, the third row gives the percentage of simulations in which both competitors have persisted to the end of simulations, and the fourth row shows simulations in which neither competitor has persisted to the end of simulations. Note that by definition the first and second rows have values of 0 when competitors have equal fitness because neither competitor can be more or less fit when there is no fitness inequality.

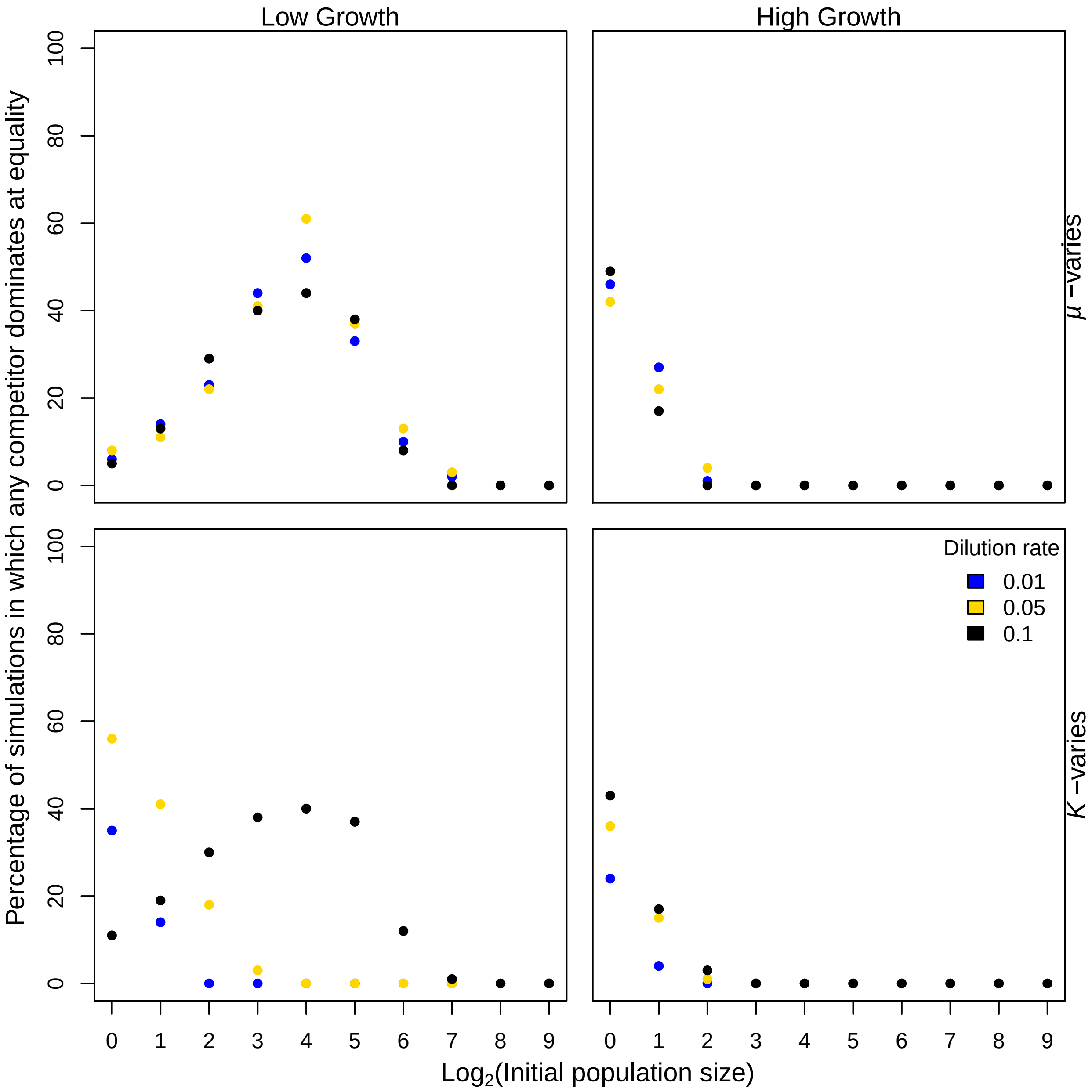

Figure S9: The percentage of simulations resulting in dominance by one competitor (either species) at fitness equality as a function of initial population size and growth rate when either μ or K is varied in the stochastic framework at three different dilution rates.

Table S1: Listing of all the different models we examined to assess the generality of the results.

Dilution rate	Parameter varied	Growth rate	Deterministic/Stochastic framework
0.01	μ	Low	Stochastic
0.05	μ	Low	Stochastic
0.10	μ	Low	Stochastic
0.01	μ	High	Stochastic
0.05	μ	High	Stochastic
0.10	μ	High	Stochastic
0.01	K	Low	Stochastic
0.05	K	Low	Stochastic
0.10	K	Low	Stochastic
0.01	K	High	Stochastic
0.05	K	High	Stochastic
0.10	K	High	Stochastic
0.01	μ	Low	Deterministic
0.05	μ	Low	Deterministic
0.10	μ	Low	Deterministic
0.01	μ	High	Deterministic
0.05	μ	High	Deterministic
0.10	μ	High	Deterministic
0.01	K	Low	Deterministic
0.05	K	Low	Deterministic
0.10	K	Low	Deterministic
0.01	K	High	Deterministic
0.05	K	High	Deterministic
0.10	K	High	Deterministic
